# A randomised placebo-controlled study of the effects of lysergic acid diethylamide microdosing (15 μg) on pain perception in healthy volunteers

**DOI:** 10.1177/20494637251371626

**Published:** 2025-09-04

**Authors:** Mauro Cavarra, Nadia R. P. W. Hutten, Jan Schepers, Natasha L. Mason, Eef L. Theunissen, Matthias E. Liechti, Kim P. C. Kuypers, Valerie Bonnelle, Amanda Feilding, Johannes G. Ramaekers

**Affiliations:** 1 Department of Neuropsychology & Psychopharmacology, Faculty of Psychology & Neuroscience, Maastricht University, Maastricht, The Netherlands; 2 Department of Methodology and Statistics, Faculty of Psychology and Neuroscience, Maastricht University, Maastricht, the Netherlands; 3 Division of Clinical Pharmacology and Toxicology, Department of Biomedicine and Department of Clinical Research, University Hospital Basel, University of Basel, Basel, Switzerland; 4 The Beckley Foundation, Oxford, UK

**Keywords:** LSD, pain, microdosing, psychedelics

## Abstract

**Background:**

Preliminary research indicates that psychedelics may hold promise as analgesic agents. This study investigated the potential analgesic effects of lysergic acid diethylamide (LSD) microdosing on pain tolerance and subjective pain perception in healthy participants.

**Methods:**

Utilizing a randomised, placebo-controlled design, participants received 15 μg of LSD or placebo over four administrations. Pain tolerance was assessed using the Cold Pressor Task (CPT), along with subjective ratings of painfulness, unpleasantness, and stress.

**Results:**

No analgesic effects of LSD were found on any of these measures in the whole sample. LSD increased blood pressure and subjective ratings of drug experience on administration days. Blood pressure was positively correlated to pain tolerance in the LSD group, whereas subjective drug experience was not. To explore whether the absence of analgesic effects of LSD could be explained by ceiling effects observed in CPT performance, post-hoc analyses were conducted in a smaller subsample of individuals that did not show ceiling effects at baseline. This post-hoc analysis suggested that LSD increased pain tolerance and reduced unpleasantness, but only after the first dose.

**Conclusions:**

Overall, the present study provided no evidence for analgesic effects of 15 µg LSD. Post-hoc analyses only revealed a marginal analgesic effect of LSD in a subsample of participants. The dose used in this study may be below the threshold dose that is needed to produce a solid and consistent analgesic effect. Future research with larger, appropriately selected samples and higher doses is recommended to further elucidate LSD’s analgesic effects and its application in clinical settings.

## Introduction

Classic psychedelics are a class of psychotropic drugs that cause marked changes to consciousness through their agonist action on serotonergic receptors^1^ and have come to the attention researchers for their potential in the treatment of psychiatric conditions such as treatment-resistant depression (TRD),^2^ and addiction,^3,4^ anxiety and depression in end-of-life settings.^5,6,7^

Another area of potential clinical interest is the treatment of painful conditions that are refractory to current therapies. Retrospective studies show that cluster headache patients report relief after psychedelic use in both high and lower doses.^8,9^ More recently, a survey study in chronic pain patients showed that the reported improvements were greater than those achieved with conventional medication.^10^ Also users of low doses report higher effectiveness in the treatment of a variety of medical complaints compared to conventional medication.^11^ Supporting evidence was provided by a small sample (*n* = 10), double-blind, placebo-controlled, crossover study in migraine patients. Compared to baseline, participants who took two doses of psilocybin (10 mg) 2 weeks apart had a greater reduction in the number of attacks than the placebo group in the 2 weeks following the last drug administration.^12^

The analgesic potential of lysergic acid diethylamide (LSD) in end-of-life settings and in phantom limb pain was supported by the first wave of psychedelic research (i.e. a period during the 1960s in which considerable scientific interest arose around the effects of psychedelic drugs).^13–16^ More recently, a healthy participants study showed that a low LSD dose (20 μg) increased tolerance to experimentally-induced pain and reduced pain and unpleasantness ratings compared to placebo, with only mild effects on consciousness.^17^ If low, non-hallucinogenic doses (i.e. doses that do not cause marked alteration in the state of consciousness) of psychedelics produce analgesic effects and if these effects extend to chronic pain patients, they may be suitable future treatment options for pain patients.

Microdosing is a practice that involves taking low psychedelic doses to self-medicate, improve psychological well-being, physical and/or cognitive performance.^10,11^ While there is no consensus on physiological criteria to determine whether a psychedelic dose is a microdose, individuals engaging in microdosing usually take about 1/10 of the conventional full dose (e.g. 15 μg in the case of LSD).^10,11^ While no controlled study has been conducted to test the analgesic potential of a repeated microdosing regimen, the practice has become increasingly popular and survey studies indicate that individuals engaging in microdosing report beneficial effects on pain.^11^ This study aims at testing the potential of a LSD microdosing schedule to improve pain tolerance in a healthy sample. We hypothesized that a microdosing schedule of 15 μg of LSD taken twice a week would increase pain tolerance and reduce ratings of pain, unpleasantness, and stress during exposure to a pain-evoking task. The study by Ramaekers et al. (2021) testing the analgesic effects of LSD found that 20 μg of LSD was effective while 10 μg was not.^17^ Since 20 μg also produced noticeable (albeit small) changes in consciousness,^17^ a dose of 15 μg LSD was chosen to minimise subjective, psychedelic effects while aiming to retain analgesic effects. Finally, considering that the increase in blood pressure (BP), heart rate (HR), and the subjective effects generated by LSD may be associated with pain experience,^17^ we sought to test whether such relationships also existed within the present sample. Such investigation is relevant as, if an analgesic effect were detected, it would provide grounds to justify a larger study on clinical populations suffering from chronic pain.

## Methods

### Trial design

The study was conducted according to a randomised, double blind, placebo-controlled, parallel group design including two treatment groups; one receiving microdoses of LSD (15 μg) two times a week for 2 consecutive weeks (4 doses in total) and one receiving a placebo according to the same treatment schedule.

### Participants

Inclusion criteria were: proficient use of the English language and a body mass index between 18 and 28. This latter criterion was included to target healthy volunteers and to prevent potential effects of excess adipose tissue and altered liver metabolism leading to differences in absorption, distribution and clearance of drugs. Exclusion criteria included use of psychotropic medications (e.g. antidepressants, anxiolytics, antipsychotics), exposure to a psychedelic drug (e.g. LSD, psilocybin, ayahuasca/dimethyltryptamine) in the past 3 months, history of drug addiction, use of any psychoactive substances (including psychedelics, sedatives, cannabinoids, and stimulants) during the study, previous experience of serious side effects to psychedelic drugs, pregnancy or lactation, history of psychiatric disorders, and family history of psychotic disorders. Data collection was performed in the psychopharmacology labs at Maastricht University.

Prospective participants were informed about the procedures and risks associated with the study during a video-call with one of the researchers during which they were encouraged to ask any question. If they still wanted to participate, they were asked to sign the informed consent and then invited to a screening visit. The study was conducted according to the code of ethics on human experimentation established by the declaration of Helsinki^18^ and amended in Fortaleza, in accordance with the Medical Research Involving Human Subjects Act (WMO) and was approved by the Academic Hospital and University’s Medical Ethics committee. All participants were fully informed about all procedures, possible adverse reactions, legal rights and responsibilities, expected benefits, and their right to voluntary termination without consequences. The study was registered in the Netherlands Trial Register (Trial NL70508.068.19) and the Dutch Central Committee on Research Involving Human Subjects trial registry (NL-OMON55178). Participants in the study were financially compensated for their invested time according to the guidelines issued by the Ethics Committee (i.e. 10€/h; 355€ in case of full completion).

### Interventions

LSD base (15 μg; LSD base refers to the pure, non-salt form of lysergic acid diethylamide, as opposed to commonly used salts like LSD tartrate) was formulated as an oral solution in 0.6 mL 96% ethanol, according to good manufacturing practices.^19^ Placebo consisted of a 0.6 mL ethanol solution only.

Prior to the first study day, participants were medically screened (medical history, psychiatric history, pregnancy test) to ensure adherence to the inclusion and exclusion criteria and, if cleared, were then familiarised with tests and study procedures (i.e. they were shown the testing room, they went through a trial run of the CPT and they were shown the VAS scales that will be used during the study). Participants were instructed to abstain from recreational drug use for at least 7 days before the start of the study and throughout its duration. Additionally, they were required to avoid alcohol consumption for at least 24 h before and on the study days. These measures were taken to minimise the risk of drug interactions. They were also instructed not to consume caffeinated or alcoholic beverages on test days and the evening before, and to arrive well-rested at the test facility. On arrival, participants were screened for the presence of drugs (THC, opioids, cocaine, amphetamine, 3,4-Methylenedioxymethamphetamine) and alcohol through urine tests. An additional pregnancy test was given to female participants. If all tests were found to be negative, participants were allowed to proceed.

**Figure 1.**
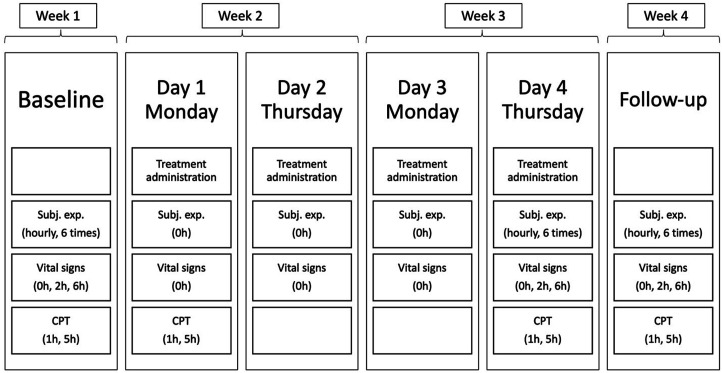
Test days lasted 6.5-h except for test days 2 and 3 which lasted 1.5 h.

Participants were invited to the laboratory for a baseline assessment, and 1 week later, the treatment began. LSD microdoses were administered on Mondays and Thursdays at 10 AM for the following 2 weeks. On the first and fourth dose of LSD pain tasks were administered to assess the effects of microdosing at the beginning and end of the schedule. These pain tasks were conducted 1 h and 5 h after administration to evaluate the stability of potential effects throughout the day. Participants were accompanied by assistants at all times or waited nearby and checked on them regularly (i.e. participants were asked how they were doing and if they needed anything between the administration of tests). In breaks, participants could use electronic devices, study or read. To test for potential long-lasting effects of LSD microdosing on pain tolerance, an identical test day without treatment administration was held in week 4 (Figure 1). This frequency and structure of administration was chosen to reflect current practices in the microdosing communities.^20^ A complete schedule of study and dosing days is provided in Figure 1. On test days in which pain tasks were not included, participants left the lab right after administration.

### Outcomes

#### Pain measures

The Cold Pressor Task (CPT)^21^ – a well-established, safe, consistent, and controllable method to induce pain^22^ – was used to induce a painful sensation. Participants were asked to keep the dominant hand submerged in cold water (between 2.5°C and 3°C) for as long as possible. The water temperature was measured just before the immersion to guarantee the reproducibility of the test. Participants were informed that the procedure could be painful and that they could stop at any time. The maximum duration of immersion (pain tolerance) was set at 180 s and in case the maximum time was reached, the researcher would instruct participants to remove their hand from the water. Participants were unaware of the time limit and the duration of immersion was used as a measure of pain tolerance, the primary outcome of this study. The primary hypothesis was that participants in the LSD group would show higher pain tolerance compared to the placebo group on treatment days. Immediately after the end of the test, participants were asked to rate painfulness, unpleasantness, and stress during the task on a visual analogue scale (VAS). These were set as secondary outcomes for this study and it was expected to observe lower levels of painfulness, unpleasantness, and stress in the LSD group compared to the placebo group on treatment days. The VAS scales were presented as 100 mm long horizontal lines marked with ‘not at all’ on the left and ‘extremely’ on the right. CPT was performed at 1h and 5h post-treatment on test days 1 and 4, and at similar timepoints at baseline and follow-up.

#### Subjective experience

On dosing days 1 and 4, before drug administration and every hour thereafter, participants were presented with a VAS asking: ‘How much do you feel under the influence of the treatment?’ It was presented as a 100 mm long horizontal line marked with ‘not at all’ on the left and ‘extremely’ on the right.^23^ On other test days, subjective experience was only recorded before administration.

#### Vital signs

On days in which the pain task was administered, before administration, 2 h and 6 h after administration BP and HR were collected. On other test days, subjective experience was only recorded before administration.

#### LSD concentration in blood plasma

Blood samples were taken 2 h after treatment administration. The blood was centrifuged, and pipetted plasma was frozen at −20°C until analysis. LSD and O-H-LSD concentrations were determined using ultra high-performance liquid chromatography-tandem mass spectrometry (UHPLC–MS/MS) as previously described^19^ in order to confirm the presence of LSD in blood at the time of testing.

### Sample size

The primary hypothesis implies an interaction effect between treatment (LSD vs placebo) and time (baseline and follow-up measures). GPower^24^ was used to determine the sample size needed for an F-test of within-between interaction in a repeated measures ANOVA to have 80% power – using alpha = 0.05 – for detecting an effect size f = 0.25, assuming a default correlation of +0.5 among repeated measures. This analysis yielded a total required sample size of N = 24. Note that the data analyses were not conducted by means of repeated measures ANOVA (see Statistical methods) but by Linear Mixed Model (LMM) analysis because the latter does not imply listwise deletion of participants if there are any missing observations. Note that the chosen effect size for this sample size calculation corresponds to a small-to-moderate effect according to Cohen’s conventions and it is based on prior work in the field reporting small-to-medium effects of low-dose psychedelics on pain measures.

### Randomisation

A block randomisation with groups of *n* = 4 was built in, so that the maximum difference between the intervention and control group is *n* = 2 persons. This randomisation was done by one experimenter who was not involved in the study and did not come in direct contact with the subjects.

### Blinding

The study treatment was blinded to the subject and the respective researcher. One experimenter who was not involved in the study and did not come into contact with the participants prepared the treatments according to group allocation.

### Statistical methods

To investigate the effects of treatment on pain outcome measures, statistical analysis was conducted with IBM SPSS Statistics (Version 26) using a Linear Mixed Models (LMM) analysis. The model included Fixed effects for Treatment (placebo vs LSD), Test day (baseline, treatment day 1, treatment day 4, follow-up), Time (1h and 5h post-administration) and Treatment × Test day, Treatment x Time, Test day × Time, and Treatment × Test day × Time interactions. The inclusion of these interaction effects aimed at assessing both the immediate and cumulative effects of LSD microdosing on pain tolerance across time. A random factor Subject was included and an unstructured covariance matrix was used. Given that the study included a baseline measurement, statistical evidence for a treatment effect would appear as a significant Treatment x Test day interaction or as a Treatment × Test day × Time interaction. If these significant interaction effects were detected, further single-degree-of-freedom interaction contrasts were conducted.

Since variations in BP and HR were shown to have an effect on pain experience in a previous study with acute doses of LSD,^17^ we ran additional LMM analyses including vital signs measures as covariates to test their potential role as mediators, in case evidence for an effect of treatment was found. Since the intensity of subjective effects of LSD was also shown to be associated with pain perception,^17^ we ran additional LMM analyses including the under the influence score as covariate to test its potential role as mediator. To test the association between vital signs and pain measures, non-parametric Spearman correlations between systolic BP, diastolic BP, HR, and pain tolerance were tested. Additionally, canonical correlation analyses between a first canonical variable composed by measures of vital signs (i.e. systolic BP, diastolic BP, and HR) and a second one composed of all pain measures (i.e. pain tolerance, painfulness, unpleasantness, and stress) were also tested.

To test the effects of 15 μg of LSD on subjective experience we ran LMM analyses on a model that included Fixed effects for Treatment (placebo vs LSD), Test day (baseline, treatment day 1, treatment day 4, follow-up), Time (0 h to 5 h post-administration), and Treatment x Test day, Treatment × Time, Test day × Time, and Treatment × Test day × Time interactions and the under the influence scale as dependent variable. A random subject intercept was included to account for the dependency between the daily measurements (i.e. factor Time) and an unstructured covariance matrix was estimated to account for dependencies between Test days. In case a significant Treatment × Time interaction was found, the same LMM was run on data obtained on treatment day 1 and 4 to test for potential effects related to LSD tolerance building.

Analyses of pain parameters described above were also repeated in an exploratory, post-hoc analysis of analgesic effects of LSD in a subsample excluding pain resistant participants, that is, participants who kept their hand in cold water for the full 180 s at baseline or in 3 out of 4 (75%) CPT administrations on treatment days.

## Results

The study ran from January 2020 until February 2022. In total, 53 healthy adults (18 to 65 years of age; mean age 36.9, SD = 16.6), were recruited from the general population. Three participants dropped out prior or during the study entrance and two participants were excluded for which no CPT data was recorded (see supplemental material S4. CONSORT 2010 Checklist and flowchart). Datasets of *n* = 48 (24 female, 24 male) participants entered the statistical analyses. Lifetime psychedelic use (i.e. used a psychedelic at least once) was reported by 28 (58.3%) participants and 14 (29%) of them used a psychedelic at least once in the past year. In this context, the use of psilocybin was reported most frequently (*n* = 18, 38%) followed by LSD (*n* = 9, 19%) and ayahuasca (*n* = 3, 6%). The lifetime use of alcohol was reported by 45 (94%) participants. Use of cannabis (*n* = 33, 69%), cocaine (*n* = 18, 38%), ecstasy (*n* = 10, 21%), amphetamines (*n* = 8, 17%), and other substances (*n* = 5, 10%) was also reported.

The following sections will report the main findings related to the study’s hypothesis. Full results are provided in Table S1 and S2.

### LSD concentration in blood plasma

Mean (SD) concentrations of LSD 2 h after dose 1 and dose 4 were 302 (105) pg/mL and 326 (117) pg/mL, respectively. Mean (SD) concentrations of O-H-LSD after dose 1 and dose 4 were 17 (7.2) pg/ml and 17 (4.9) pg/mL, respectively.

### Pain tolerance

**Figure 2.**
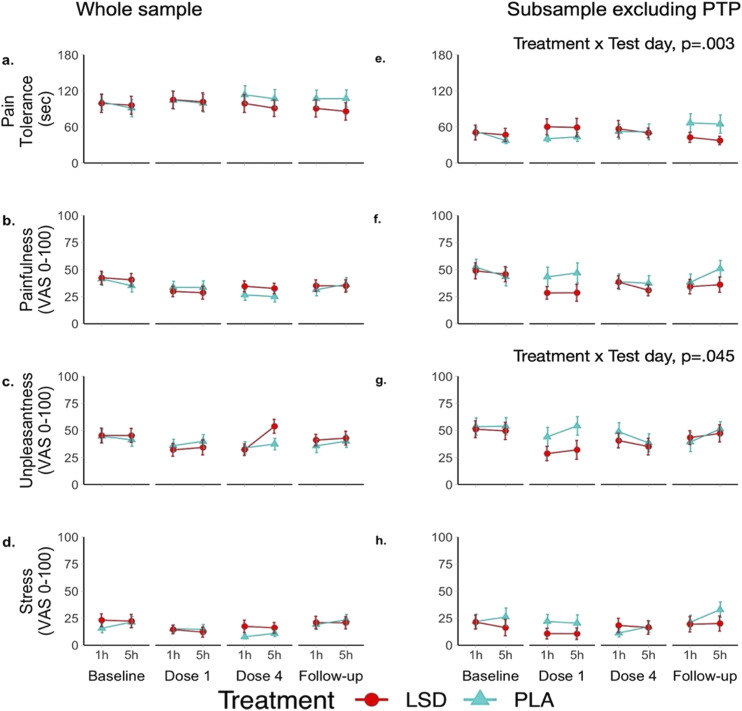
Mean pain (SE) tolerance (a and e) and subjective ratings of painfulness (b and f), unpleasantness (c and g), and stress (d and h) as a function of treatment group, test day and time in the whole sample and the subsample obtained by excluding pain tolerant participants (i.e. participants in both treatment groups who were pain tolerant at baseline or during 3 out of 4 CPT administrations on treatment days).

Means (SE) for pain tolerance, unpleasantness, pain intensity, and stress are shown in Figure 2. LMM revealed no significant Treatment x Test day (i.e. whether the change in outcomes across the different test days differs between the groups; *p = .198*) nor a Treatment x Test day × Time interaction (i.e. whether the difference in time-of-day effects across test days is different between the treatment groups; *p* = .959) on pain tolerance (Figure 2(a)).

### Self-rated pain, unpleasantness, and stress

LMM revealed no significant Treatment x Test day (i.e. whether the change in outcomes across the different test days differs between the groups) nor a Treatment × Test day × Time (i.e. whether the difference in time-of-day effects across test days is different between the treatment groups) interaction on self-reported painfulness (*p* = .085 and *p* = .762, respectively) (Figure 2(b)) unpleasantness (*p* = .370 and *p* = .812, respectively) (Figure 2(d)) and stress (*p* = .096 and *p* = .941, respectively).

### Vital signs

**Figure 3.**
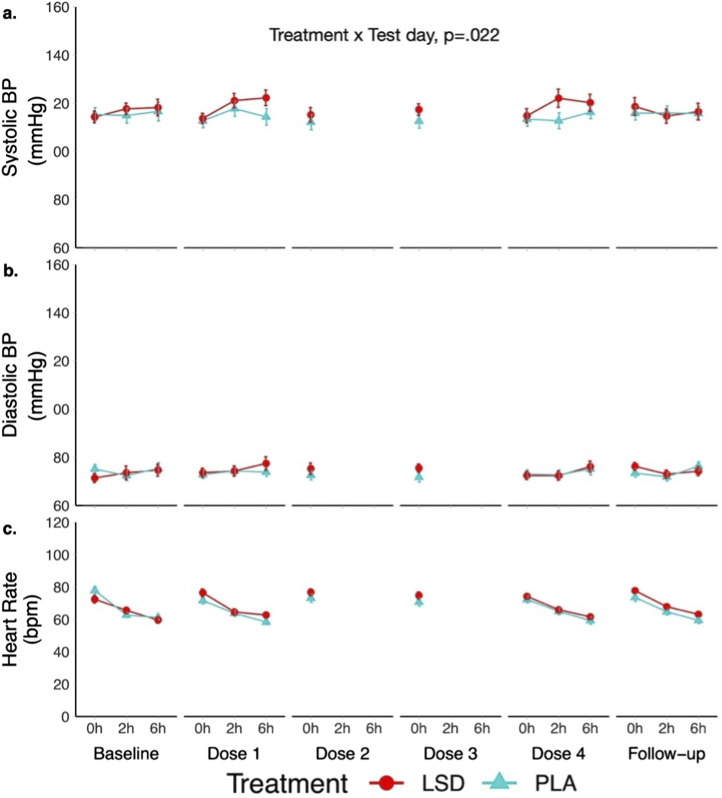
Systolic blood pressure (a), diastolic blood pressure (b) and heart rate (c) each test day, administration time and treatment group.

**Figure 4.**
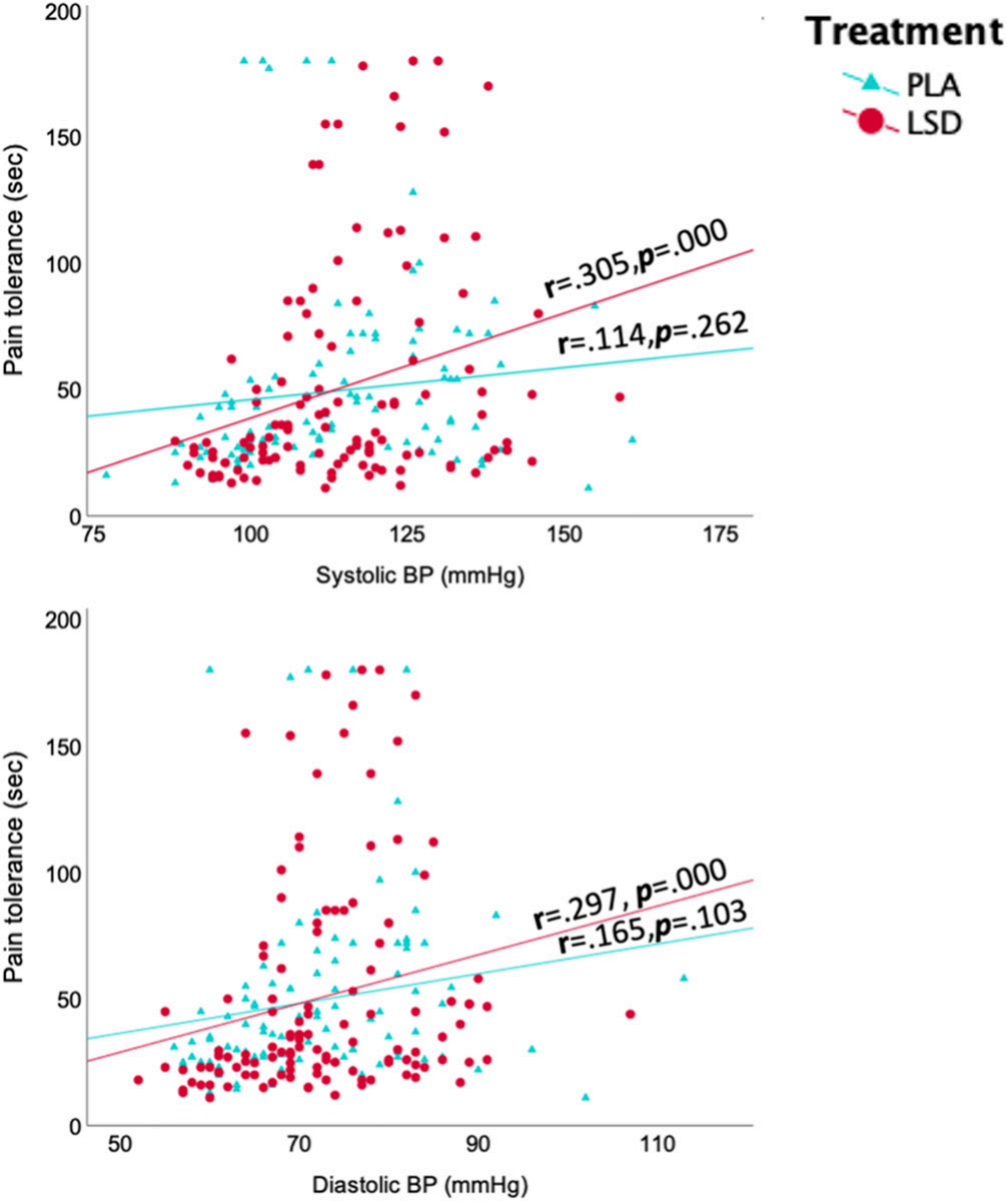
Correlations between systolic/diastolic blood pressure and pain tolerance in both treatment groups.

Variations in vital signs measures (Figure 3) all occurred within the normal range. LMM analyses showed a significant Treatment × Test day interaction on systolic BP (F (5, 234) = 2.7; *p* = .022). Spearman correlation revealed a significant association of pain tolerance with systolic (ρ = .305, *p* = .000) and diastolic (ρ = .297, *p* = .000) BP in the LSD group (Figure 4). Canonical correlation between measures of BP and HR on the one hand and pain measures on the other indicated a significant positive association (F (44, 343) = 1.94, *p* = .001, canonical r = .41) that explained 16% of the total variance. More specifically, the higher HR and BP, the stronger the analgesic effect.

### Subjective experience

**Figure 5.**
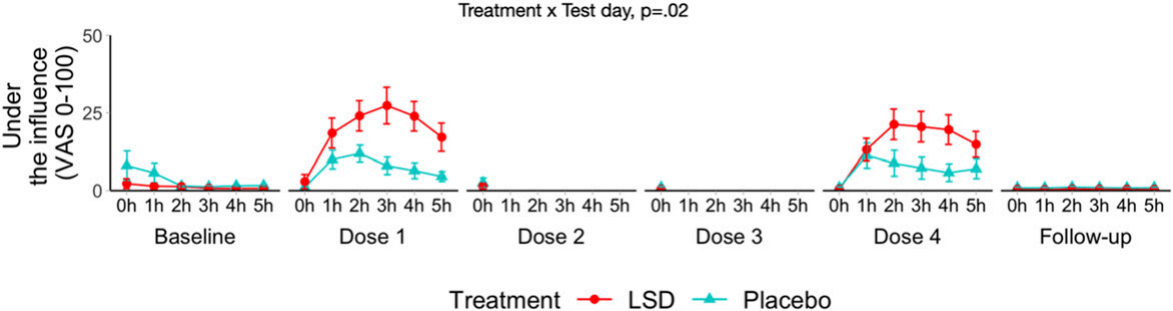
Mean (SE) ratings of ‘under the influence’ as a function of treatment, day of treatment and time to administration (***p < .001).

Mean under the influence scores are shown in Figure 5. LMM analyses showed a significant Treatment × Test day interaction (i.e. whether the change in outcomes across the different test days differs between the groups) on the under the influence score (F (3, 95) = 3.43, *p = .02*). An additional LMM utilising only data from treatment day 1 and 4 was ran to test for potential tolerance effects. This analysis only yielded a significant main effect of treatment (F (1, 48) = 4.125, *p = .048*), suggesting the absence of tolerance. Under the influence scores did not correlate with measures of pain.

### Exploratory, post-hoc analyses in a subsample

An unexpectedly high proportion of participants (42%) was able to sustain the cold water immersion for 180 s, which is the task’s maximal duration. Since such a ceiling effect may have masked potential Treatment effects, analyses were repeated after excluding pain-tolerant participants, that is, participants in both treatment groups who kept their hand in cold water for the full 180 s at baseline (*n* = 18) or during 3 out of 4 (75%) CPT administrations (*n* = 2) on treatment days. After this filtering, a subsample of *n* = 28 (14 placebo, 14 LSD; 17 female, 11 male, 20 to 64 years of age; mean age 35.64, SD = 16.4) volunteers remained.

In this subsample, LMM revealed a significant Treatment × Test day (i.e. whether the change in outcomes across the different test days differs between the groups) interaction (F (3, 55) = 5.196, *p = .003*) (Figure 2(e)) on pain tolerance. Single degree of freedom interaction contrasts revealed that the effect was mainly driven by the difference observed at follow-up (*β* = 29.47, *p = .013*). LMM revealed no significant Treatment x Test day interaction (i.e. whether the change in outcomes across the different test days differs between the groups) nor Treatment × Test day × Time interaction (i.e. whether the difference in time-of-day effects across test days is different between the treatment groups) on self-reported painfulness (*p* = .328 and *p* = .521, respectively) (Figure 2(f)) and stress (*p* = .138 and *p* = .592, respectively) (Figure 2(g)). A significant Treatment × Test day (i.e. whether the change in outcomes across the different test days differs between the groups) interaction on unpleasantness (F (3, 42) = 2.925, *p = .045*) was observed (Figure 2(h)). As a follow-up, single degree of freedom interaction contrasts were conducted but none of these comparisons turned out significant. The Treatment × Test day interactions in the LMM on measures of pain tolerance (F (3, 55) = 4.845, *p* = .005) and unpleasantness (F (3, 41) = 2.949, *p* = .044), remained significant after the inclusion of vital signs measures as covariates. Likewise, the Treatment x Test day interactions in the LMM on measures of pain tolerance (F (3, 55) = 5.186, *p* = .003) and unpleasantness (F (3, 42) = 2.974, *p* = .042) remained significant even after the inclusion of under the influence scores as covariates.

## Discussion

This study set out to test the analgesic potential of a LSD microdosing regimen on pain tolerance in a sample of healthy volunteers who were exposed to a pain evoking task. We hypothesized that a microdosing schedule of 15 μg of LSD taken twice a week would increase pain tolerance and reduce ratings of pain, unpleasantness, and stress during exposure to a pain-evoking task. The prevalence of drug use in our sample was higher than what’s reported in the general population^25^ likely because people who already used psychedelics may be more open to participate in psychedelic research. Our primary analyses on the whole sample revealed no difference between groups in pain tolerance (primary hypothesis) and subjective ratings of painfulness, unpleasantness, and stress. Exploratory, post-hoc analyses conducted on a smaller subsample that excluded pain tolerant participants in the CPT, revealed Treatment x Test day interactions, suggesting marginal analgesic effect of LSD, but only after the first dose. In this subsample analysis, there appeared to be an increase in pain tolerance in the placebo group as well during follow-up. Simple interaction contrasts, however, were generally non-significant, except at follow-up. It is likely, however, that the selected subsample was too small to provide sufficient statistical power to consistently detect treatment differences with simple contrasts. It should be noted that no ‘trends’ were apparent on the fourth dosing day. This might suggest that participants had developed some tolerance^26^ to the ‘mild’ analgesic effects that were trending after the first dose. Alternatively, this might suggest that 15 µg of LSD is close to a threshold dose at which mild signs of analgesic effects might appear in some but not in others, to degrees that may fluctuate over time.

Systolic blood pressure slightly increased in the LSD group but remained within the normal range. This finding is in line with earlier studies that demonstrated LSD safety at low doses.^11,27^ Present findings also showed a positive correlation between LSD-induced variations in blood pressure and pain tolerance, suggesting that changes in blood pressure might account for the Treatment × Test day interactions observed in the post-hoc analyses of pain tolerance and unpleasantness. The association between blood pressure and analgesia was already shown in both animal and human studies.^28–30^ However, the addition of blood pressure as a covariate to the LMM models of pain tolerance and unpleasantness did not alter the significance of the original Treatment x Test day interactions. This indicates that the interaction effect was not mediated by LSD induced changes in blood pressure.

Low doses of LSD also produced slight but noticeable subjective effects. These were not correlated with pain tolerance and subjective ratings of pain under LSD. Likewise, subjective ratings of drug experience did not alter the main Treatment × Test day interactions observed for pain tolerance and unpleasantness in the subsample analysis, when added to the LMM model as a covariate. The current dose might have been too low to exert a solid analgesic effect. A previous study demonstrated analgesic effects of LSD in healthy volunteers at a 20 μg but not at a 10 μg dose, suggesting that the minimal dose for inducing a reliable analgesic effect may be closer to 20 μg of LSD or higher.^17^ But even at higher doses it still needs to be determined whether LSD may be used as a novel therapeutic tool as clinical trials in patient populations are currently missing. Even though the present findings in healthy volunteers do not provide strong support for the use of low doses of LSD in the treatment of pain, there are still some relevant research questions that need further exploration before we can come to a final evaluation. For example, it is unclear from present study whether the absence of an analgesic effect under LSD was partly due to a lack of sensitivity in the Cold Pressor Task. Hence, it would be advisable to replicate this study in a wider range of pain paradigms. Future research would also need to consider higher dose regimens with LSD (i.e. > 20 μg) in the treatment of pain, to establish whether earlier findings of analgesic properties of LSD 20 μg^17^ can be replicated. Such dosing regimens would also have to evaluate the frequency and duration of microdosing to assess the sustainability of acute or long-term treatments. Ultimately, research on the analgesic properties of LSD should be conducted in patient populations to evaluate efficacy, underlying changes in neuroplasticity,^1,31,32^ tolerance after repeated dosing,^26^ specificity for different types of chronic pain conditions,^33^ and the role of psychotherapy^34^ or other psychological pain management interventions such as mindfulness^35^ and hypnosis^36^ in combination with LSD microdosing regimens.

### Limitations

A large number of participants were pain tolerant in the CPT at baseline and throughout treatment, which made it impossible to assess the potential of LSD to induce improvements in this group. Removal of these participants from the statistical analysis resulted in a smaller subsample of participants that showed some improvement in subjective experience of pain after the first LSD dose, but this subsample did not provide enough power for studying the full scope of analgesic effects. Future research should make sure to adopt experimental procedures that screen suitability of participants for CPT measures at screening^37^ in order to avoid ceiling effects due to potential higher tolerance to CPT-induced pain.

### Conclusions

Overall, primary analyses in the present study provided no support for the presence of an analgesic effect of 15 µg LSD during a repeated dosing regimen. In a sub-sample of participants who were less tolerant of cold pain, a marginal analgesic effect was observed. In other words, our findings provide no support for an analgesic effect of repeated doses. Future research with larger samples, patient populations and with higher doses is recommended to further elucidate LSD’s analgesic potential and its application in clinical settings.

## Supplemental Material

Supplemental Material – A randomised placebo-controlled study of the effects of lysergic acid diethylamide microdosing (15 μg) on pain perception in healthy volunteers Supplemental Material for A randomised placebo-controlled study of the effects of lysergic acid diethylamide microdosing (15 μg) on pain perception in healthy volunteers by Mauro Cavarra, Nadia R. P. W. Hutten, Jan Schepers, Natasha L. Mason, Eef L. Theunissen, Matthias E. Liechti, Kim P. C. Kuypers, Valerie Bonnelle, Amanda Feilding, and Johannes G. Ramaekers in British Journal of Pain

## Data Availability

The data was registered on DataverseNL and can be found on the study page: A randomised placebo-controlled study of the effects of lysergic acid diethylamide microdosing (15 μg) on pain perception in healthy volunteers.*
