# Supplementary material for: A randomised placebo-controlled study of the effects of lysergic acid diethylamide microdosing (15 μg) on pain perception in healthy volunteers
Source: Br J Pain. 2025 Sep 4;19(6):434–45. doi: 10.1177/20494637251371626 (PMC12411475; doi:10.1177/20494637251371626)

A placebo-controlled study of the effects of LSD microdosing (15 μg) on pain perception in healthy volunteers

Supplementary Materials

Supplemental Table S1. LMM results for ratings of painfulness, unpleasantness, and stress on the whole sample: coefficients, df, and p-values.

Pain tolerance (sec)			
	F	df	p
Treatment	0.167	1, 48	.685
Treatment x Test day	1.585	3, 96	.198
Treatment x Time	0.054	3, 96	.817
Treatment x Test day x Time	0.101	3, 96	.959
Painfulness rating VAS (0 - 100)			
Treatment	0	1, 47	.986
Treatment x Test day	2.284	3, 87	.085
Treatment x Time	0.023	3, 88	.879
Treatment x Test day x Time	0.388	3, 88	.762
Unpleasantness rating VAS (0 - 100)			
Treatment	0.001	1, 48	.978
Treatment x Test day	1.062	3, 83	.370

Treatment x Time	1.027	3, 84	.318
Treatment x Test day x Time	0.318	3, 84	.812
Stress rating VAS (0 - 100)			
Treatment	0.113	1, 47	.739
Treatment x Test day	2.182	3, 86	.096
Treatment x Time	5.943	3, 86	.021
Treatment x Test day x Time	0.132	3, 86	.941

A placebo-controlled study of the effects of LSD microdosing (15 µg) on pain perception in healthy volunteers

Supplementary Materials

Supplemental Table S2. LMM results for ratings of painfulness, unpleasantness and stress on the selected sample: coefficient, df and p-values.

Pain tolerance (sec)			
	F	df	p
Treatment	0	1, 28	.984
Treatment x Test day	5.196	3, 55	.003
Treatment x Time	0.019	3, 56	.892
Treatment x Test day x Time	0.424	3, 56	.737
Painfulness rating VAS (0 - 100)			
Treatment	1.202	1, 26	.283
Treatment x Test day	1.178	3, 49	.328
Treatment x Time	1.105	3, 50	.304
Treatment x Test day x Time	0.761	3, 50	.521
Unpleasantness rating VAS (0 - 100)			
Treatment	0.79	1, 28	.382
Treatment x Test day	2.925	3, 42	.045

Treatment x Time	0.433	3, 42	.517
Treatment x Test day x Time	0.082	3, 42	.970
Stress rating VAS (0 - 100)			
Treatment	0.592	1, 28	.448
Treatment x Test day	1.918	3, 50	.138
Treatment x Time	3.944	3, 50	.069
Treatment x Test day x Time	0.641	3, 50	.592

A placebo-controlled study of the effects of LSD microdosing (15 µg) on pain perception in healthy volunteers

Supplementary Materials

Supplemental Table S3. Demographic information and past psychedelic use of treatment and placebo group.

Group	Mean age (SD)	n. of females (%)	Used psilocybin (%)	Used LSD (%)	Used Ayahuasca (%)
**LSD**	36.5 (15.13)	13 (54.2)	7 (29.2)	5 (20.8)	1 (4.2)
**Placebo**	37.38 (18.24)	11 (45.8)	11 (45.8)	4 (16.7)	2 (8.3)

CONSORT 2010 checklist of information to include when reporting a randomised trial*

Section/Topic	Item No	Checklist item	Reported on page No
**Title and abstract**			
	1a	Identification as a randomised trial in the title	1
	1b	Structured summary of trial design, methods, results, and conclusions (for specific guidance see CONSORT for abstracts)	1
**Introduction**			
Background and objectives	2a	Scientific background and explanation of rationale	3
	2b	Specific objectives or hypotheses	4
**Methods**			
Trial design	3a	Description of trial design (such as parallel, factorial) including allocation ratio	5
	3b	Important changes to methods after trial commencement (such as eligibility criteria), with reasons	na
Participants	4a	Eligibility criteria for participants	5
	4b	Settings and locations where the data were collected	6
Interventions	5	The interventions for each group with sufficient details to allow replication, including how and when they were actually administered	7
Outcomes	6a	Completely defined pre-specified primary and secondary outcome measures, including how and when they were assessed	8
	6b	Any changes to trial outcomes after the trial commenced, with reasons	na
Sample size	7a	How sample size was determined	10
	7b	When applicable, explanation of any interim analyses and stopping guidelines	
Randomisation:			
Sequence generation	8a	Method used to generate the random allocation sequence	10
	8b	Type of randomisation; details of any restriction (such as blocking and block size)	10
Allocation concealment mechanism	9	Mechanism used to implement the random allocation sequence (such as sequentially numbered containers), describing any steps taken to conceal the sequence until interventions were assigned	10
Implementation	10	Who generated the random allocation sequence, who enrolled participants, and who assigned participants to interventions	10
Blinding	11a	If done, who was blinded after assignment to interventions (for example, participants, care providers, those	11

		assessing outcomes) and how	
	11b	If relevant, description of the similarity of interventions	
Statistical methods	12a	Statistical methods used to compare groups for primary and secondary outcomes	11
	12b	Methods for additional analyses, such as subgroup analyses and adjusted analyses	12
**Results**			
Participant flow (a diagram is strongly recommended)	13a	For each group, the numbers of participants who were randomly assigned, received intended treatment, and were analysed for the primary outcome	12
	13b	For each group, losses and exclusions after randomisation, together with reasons	12
Recruitment	14a	Dates defining the periods of recruitment and follow-up	12
	14b	Why the trial ended or was stopped	Na
Baseline data	15	A table showing baseline demographic and clinical characteristics for each group	S3
Numbers analysed	16	For each group, number of participants (denominator) included in each analysis and whether the analysis was by original assigned groups	12
Outcomes and estimation	17a	For each primary and secondary outcome, results for each group, and the estimated effect size and its precision (such as 95% confidence interval)	13-15
	17b	For binary outcomes, presentation of both absolute and relative effect sizes is recommended	Na
Ancillary analyses	18	Results of any other analyses performed, including subgroup analyses and adjusted analyses, distinguishing pre-specified from exploratory	14
Harms	19	All important harms or unintended effects in each group (for specific guidance see CONSORT for harms)	Na
**Discussion**			
Limitations	20	Trial limitations, addressing sources of potential bias, imprecision, and, if relevant, multiplicity of analyses	18
Generalisability	21	Generalisability (external validity, applicability) of the trial findings	18
Interpretation	22	Interpretation consistent with results, balancing benefits and harms, and considering other relevant evidence	16-18
**Other information**			
Registration	23	Registration number and name of trial registry	2, 21
Protocol	24	Where the full trial protocol can be accessed, if available	2
Funding	25	Sources of funding and other support (such as supply of drugs), role of funders	none

*We strongly recommend reading this statement in conjunction with the CONSORT 2010 Explanation and Elaboration for important clarifications on all the items. If relevant, we also recommend reading CONSORT extensions for cluster randomised trials, non-inferiority and equivalence trials, non-pharmacological treatments, herbal interventions, and pragmatic trials. Additional extensions are forthcoming: for those and for up to date references relevant to this checklist, see www.consort-statement.org.

CONSORT FLOWCHART

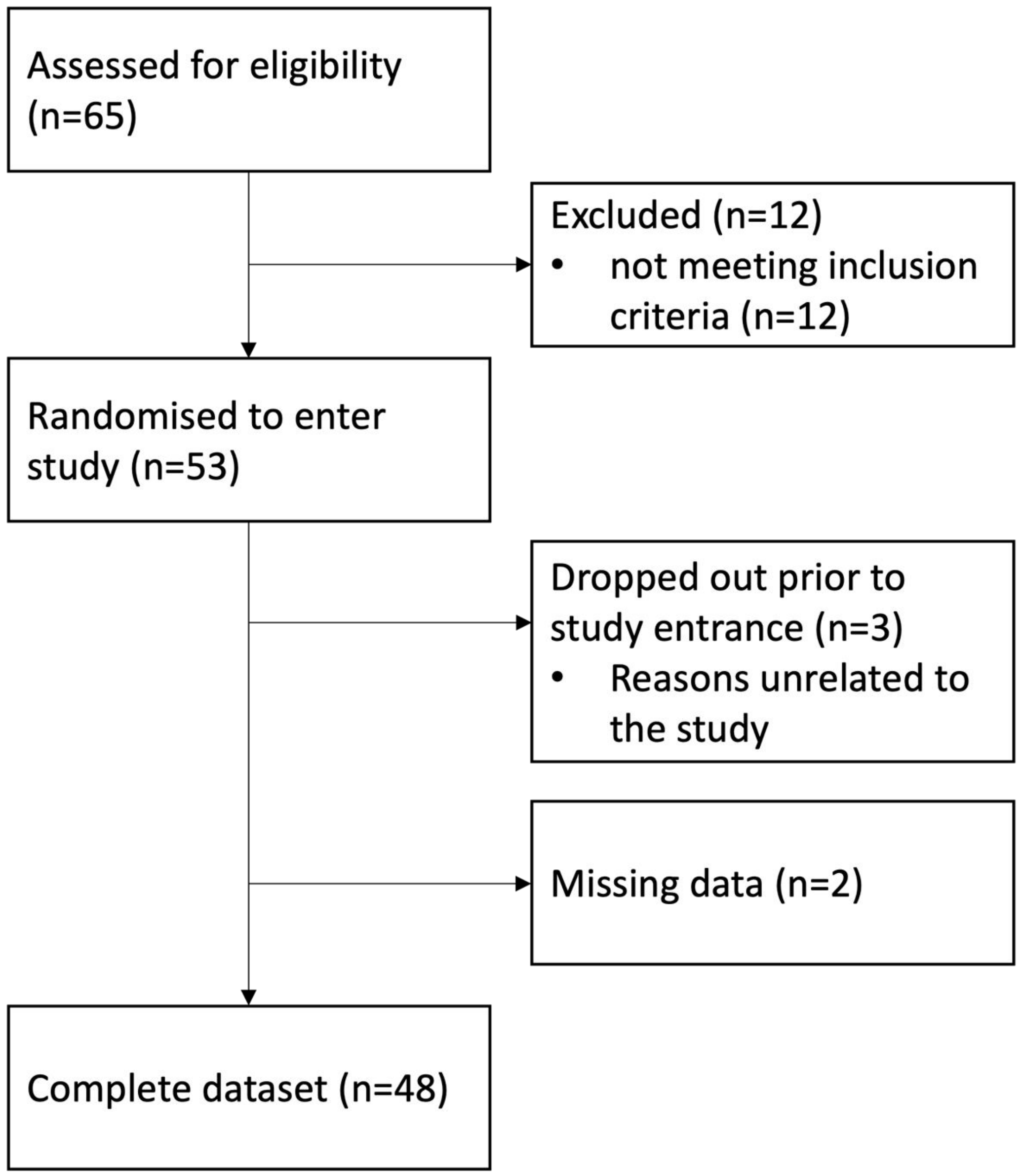

Supplement: Supplementary file 1 [file sj-pdf-1-bjp-10.1177_20494637251371626.pdf]
